# Substance Use in Sports: A Cross‐Sectional Study Among Professional Footballers in Accra, Ghana

**DOI:** 10.1002/hsr2.71828

**Published:** 2026-02-18

**Authors:** Isaac Odei, Yaw Akye Essuman, Angela Lamensdorf Ofori‐Atta

**Affiliations:** ^1^ University of Ghana Medical School Accra Ghana; ^2^ Lions Eye International Center Korle Bu Teaching Hospital Accra Ghana

**Keywords:** alcohol, cannabis, footballers, Ghana, nicotine

## Abstract

**Background and Aims:**

Substance use in sports, though harmful, is common but with little evidence from Africa. This study aims to assess the prevalence and self‐reported motivations for substance use, as well as to characterize the patterns of alcohol, tobacco, and cannabis use among professional footballers in Accra.

**Methods:**

This cross‐sectional study included 139 professional footballers from nine teams competing in the Ghana Premier League and Division One League within the Greater Accra Region. Participants were selected using convenience sampling. Substance use was assessed with the Alcohol Use Disorders Identification Test (AUDIT), Fagerström Test for Nicotine Dependence (FTND), and Cannabis Use Disorders Identification Test–Revised (CUDIT‐R) to evaluate alcohol, tobacco, and cannabis use patterns, respectively.

**Results:**

The prevalence of substance use was 44.6%, with caffeine prevalence highest (30.2%), followed by alcohol (15.8%), 2.9% for cannabis, and 2.2% for tobacco. The mean AUDIT score amongst alcohol users was 3.6 ( ± 3.7), representing low‐risk consumption. The mean CUDIT‐R score was 6.3 ( ± 4.6), consistent with low‐risk cannabis use, while the mean FTND score was 0 ± 0, reflecting low nicotine dependence. Pleasure was the most frequently reported reason for alcohol, cannabis, and tobacco use, whereas energy enhancement was the main reason for caffeine use. Knowledge of performance‐enhancing drugs was low (2.2%), and no participant reported their use. A negative association was observed between the number of years spent at the current club and substance use (OR = 0.81; 95% CI 0.65–0.99; *p* = 0.042).

**Conclusions:**

Participants in this study reported high usage of caffeine and low usage of alcohol, cannabis, and nicotine. There was also low dependence on these substances. Pleasure or recreational use was the commonest reason for substance use. Caffeine was most popularly used for energy. Health education, counselling, and scheduled substance use monitoring of athletes are necessary to prevent inadvertent use, maintain low levels and prevent progression to high‐risk use.

## 1 Introduction

Substance use continues to attract global attention because of its significant impact on health and quality of life. An estimated 35 million people worldwide suffer from drug use disorders, and athletes are not exempt [1]. Unlike in the general population, where substance use often centers on recreational or dependence‐driven consumption, athletes may engage in drug use both for performance enhancement (PEDs) and as a coping mechanism. Studies show that sportsmen and women in both team and individual sports use substances such as anabolic steroids, alcohol, opioids, and stimulants [2, 3, 4]. The International Olympic Committee (IOC) reports several negative effects of substance use in sports, including dehydration, delayed healing, hypertension, and impaired reaction times. Cannabis use has been linked to poor coordination and reaction, while caffeine use may cause anxiety and insomnia [5].

Substance use exists along a spectrum—from recreational use to abuse and dependence. Substance abuse typically involves the harmful or hazardous use of substances, including prescription medications for non‐medical purposes. Dependence, on the other hand, is characterized by compulsive use despite adverse consequences, cravings, impaired control over intake, and withdrawal symptoms upon cessation. Given these potential trajectories and their implications for sporting performance, several global initiatives have been established to safeguard athlete health and the integrity of sports. Notably, the World Anti‐Doping Agency (WADA) and the World Anti‐Doping Code provide frameworks to prevent and manage substance use among athletes [6]. Additional preventive strategies include athlete education, biochemical testing of urine and blood samples, and integration of mental health professionals within sports teams for screening, treatment, and follow‐up [7, 8].

Alcohol use remains a major contributor to global morbidity and mortality [9]. Its diuretic properties can significantly compromise cardiovascular endurance in athletes [10], while its negative impact on glucose metabolism leads to reduced muscle performance [11]. Nicotine is sometimes used among athletes for appetite suppression and pre‐match preparations [12]; however, young users are particularly susceptible to rapid nicotine dependence [13]. Anabolic agents, when combined with strength training, can increase fat‐free mass, muscle size, and power [14], yet chronic or excessive use may precipitate manic‐like symptoms, impulsive behavior, and increased risk of sports injury [15]. Cannabis is often used for its anxiolytic, sedative, and analgesic properties [16, 17], but may impair risk perception, coordination, and movement, i.e., factors that can detrimentally affect athletic performance [18]. Similarly, caffeine, though commonly used to delay fatigue, has been associated with binge drinking and other risky behaviors [19, 20].

The growing participation of young Africans in competitive sports heightens their vulnerability to substance use [21, 22]. Emerging studies from Africa indicate varying prevalence rates across substances and countries: 41% for alcohol among Cameroonian footballers [23], 25% for marijuana among Nigerian footballers [24], and 37% for caffeine use among Malawian players [25]. Additionally, one‐third of Nigerian amateur footballers reportedly mix alcohol with energy drinks [25], a practice that can be hazardous as caffeine may mask the intoxicating effects of alcohol, fostering a false sense of control and promoting risky behaviors.

In Ghana, substances commonly used in the general population include alcohol, tobacco, methamphetamine, and opioids [26, 27]. The reported prevalence of alcohol and marijuana use among Ghanaian youth stands at 12% and 16.2%, respectively [28]. Furthermore, about one‐third of smokers have been found to exhibit high or very high dependence on cigarettes [29]. Although few studies have explored substance use among Ghanaian athletes, existing evidence points to possible use even among non‐professional sports participants. The use of anabolic–androgenic substances, though relatively low, has been documented among Ghanaian high school athletes [30]. Reports also indicate the use of tramadol, energy drinks, and cannabis‐infused confections (“marijuana toffee”) among student athletes to enhance performance or control weight [31]. Despite high awareness of WADA‐prohibited substances among elite university athletes, knowledge of the specific health effects of these drugs remains limited, except for cannabis [32].

Recreational and professional football remain among the most popular sporting activities in Ghana, engaging large numbers of young people and requiring efforts to ensure safe, health‐promoting environments. However, at the time of this study, no published research had investigated the burden of substance use among professional footballers in the country. In addition, there was very little data on the subject in African football and sports broadly. Furthermore, while two previous African studies examined motivations for substance use without quantifying the extent of use [25, 33], others did not assess either the prevalence or underlying reasons [23, 24].

The study therefore, aimed to determine the prevalence of substance use among professional footballers in Accra, assess the extent of use and risk of dependence, and explore the underlying motivations for substance use. Findings from this work will provide valuable insights into the burden of substance use among professional athletes, identify priority areas for prevention and control, and inform health policies and interventions designed to protect athletes' well‐being and uphold the integrity of sports.

## 2 Materials and Methods

This was a descriptive cross‐sectional study that took place between February to August 2024. Study participants included registered footballers from 7 clubs in the Ghana Premier League and 4 clubs in the Ghana Division 1 League, both male professional leagues. We included all players registered with the aforementioned leagues and excluded all who declined to enrol in the study or who lived outside the Greater Accra Region.

Using a prevalence rate of 91.0% from a similar study [33], the Cochran's formula [34] was used to derive a minimum sample size of 126 participants at a 95% confidence level and 5% margin of error. All 11 Accra‐based teams were contacted through their respective team managers, with all agreeing to participate, and each team was expected to have 33 players for systematic sampling. At the time of data collection, however, only nine out of eleven teams were available. In addition, there were varying numbers of players in each team, ranging from 14 to 20, who were available to participate. This variation rendered systematic sampling impractical as the sampling interval would have resulted in fewer players per team than was required to achieve the minimum sample size. All available and consenting players in the teams were therefore included in the study.

After ethical clearance was given by the University of Ghana Community Health Dissertation Review Committee (UGMS‐CHDRC/152/2024) and approval was sought from teams, the players were approached and the study was explained to them. Written informed consent was obtained, and an interviewer administered the questionnaire to consenting players. Assent was obtained for minors. Players were interviewed in turn and privately by the principal investigator and without the presence of any authority figures such as coaches, team managers, or captains. Interviewer‐administered questionnaire was adopted as it guaranteed more responses, helped to clarify any unclear questions, facilitated rapport building, and was better suited for players with low literacy.

The data collection tool was a questionnaire, developed as follows: An extensive review of substance use literature in sports was conducted under the supervision of experts to determine substances used in sports, whilst keeping local nuances in mind. Internationally recognized tools for measuring dependence of the substances were then identified and evaluated for their appropriateness ‐ length, reliability, and language appropriateness. Alcohol Use Disorders Identification Test‐10 (AUDIT), Cannabis Use Disorders Identification Test‐Revised (CUDIT‐R), and Fagerström Test for Nicotine Dependence (FTND) instruments for alcohol, cannabis, and tobacco, respectively, were selected to measure the extent of use. Given the absence of locally validated tools and the under‐researched nature of substance use among African athletes, these standardized instruments provided a rigorous and comparable framework for assessing substance use patterns. The final tool was reviewed by experts and pre‐tested in a cohort of Ghanaian university youth for linguistic clarity and cultural appropriateness before administration.

The AUDIT is an internationally recognized ten‐item screening tool developed by the World Health Organization (WHO), designed for use in primary health care settings to identify harmful, hazardous, and likely dependent drinkers. Questions cover frequency, quantity, and drinking consequences. Each answer is assigned a score, from 0 to 4 for the first eight questions and 0, 2, or 4 for the last two questions. The total score obtained by a respondent is compared to a predetermined WHO set of cut‐offs. The maximum score is 40, with a score of 1 to 7 suggesting low risk consumption, 8 to 14 ‐ hazardous or harmful consumption ‐ and 15 or more indicating likelihood of alcohol dependence (moderate to severe alcohol use disorder) [35].

The CUDIT‐R, a revised version of the Cannabis Use Disorders Identification Test (CUDIT), is a screening tool validated in general population samples as well as adolescent and young adult samples for measuring cannabis misuse [36]. It is composed of 8 items, assessing: consumption of cannabis, physical dependence, and psychological features. Scores range from 0 to 32, with a cut‐off score of 7, above which is hazardous use, and 13 suggestive of a probable DSM‐IV diagnosis of CUD (dependence) [37].

The Fagerström Test for Nicotine Dependence (FTND) is a standard instrument for assessing the intensity of physical addiction to nicotine. Designed to provide an ordinal measure of nicotine dependence as pertains to cigarette smoking, it contains six items that evaluate the quantity of cigarette consumption, the compulsion to use, and dependence. In scoring the test, yes/no questions are scored from 0 to 1, whereas multiple‐choice questions are scored from 0 to 3. A total score of between 0 and 10 is given. Scores from 7 to 10 indicate high dependence, 4–6, moderate dependence, and < 4, minimal dependence [38].

Although these instruments (AUDIT, CUDIT‐R, and FTND) have not yet been formally validated in Ghanaian populations, they have demonstrated acceptable properties and been successfully deployed across diverse international and African contexts, including Nigeria, South Africa, and Tanzania. The AUDIT has shown good reliability and validity among university students in Nigeria [39] and community samples in Tanzania [40], while the CUDIT‐R and FTND have been applied successfully in South African populations [41, 42].

Section A gathered relevant sociodemographic characteristics such as age, level of education, secondary occupation, and religion of participants. Section B recorded details of their football career, including: length of football career, position they played, and length of stay at current club. Section C assessed the types of substances used by participants, as many as applied, and their extent of use. Pattern of use for alcohol, tobacco, and cannabis was determined by the AUDIT, CUDIT‐R, and FTND instruments, respectively. Section D investigated the self‐reported motivations for substance use, as many as applied.

Data entry and analysis were done using Statistical Package for Social Sciences (SPSS) Version 25 (IBM Corp., Armonk, NY, USA). Demographic characteristics, prevalence, and factors influencing use were analyzed by descriptive statistics and reported as means, percentages, and frequencies. Standard deviation was provided for mean AUDIT, CUDIT‐R, and FTND scores. Each participant's score on each of the standard tests ‐ AUDIT, CUDIT‐R, and FTND ‐ was compared to standard cut‐offs to determine the pattern of use. Logistic regression was employed to determine the relationship between socio‐demographic and football characteristics and substance use, with *p*‐values less than 0.05 considered statistically significant.

## 3 Results

Table 1 highlights the characteristics (demographic and football career) of selected participants. All participants were male, with a median age of 20 years (15–29 years). Nearly all participants were below 25 years of age (130/139, 93.7%), with most having attained at least secondary‐level education (113/139, 81.3%). Football constituted the primary source of income for the majority of players (113/139, 81.3%), and representation across playing positions was relatively balanced.

**Table 1:** Demographic and footballing career characteristics of participants (*N* = 139).

Characteristics	Frequency (*n*)	Percentage (%)
Total	139	100.0
Sex		
Male	139	100.0
Age, years		
15–19	64	46.0
20–24	66	47.7
25–29	9	6.3
Educational level		
No formal education	1	0.7
Junior high school	25	18.0
Senior high school	95	68.3
Tertiary	18	13.0
Religion		
None	2	1.4
Christianity	107	77.0
Islam	30	21.6
Marital status		
Single	99	71.2
In a relationship	35	25.2
Married	5	3.6
Position played		
Forward	44	31.6
Midfielder	40	28.9
Defender	41	31.4
Goalkeeper	14	10.1
Source of income		
Football only	113	81.3
Football plus other source	26	18.7

### 3.1 Prevalence of Substance Use

Table 2 presents the self‐reported use of substances among participants. The prevalence of substance use in this study was 44.6% (62/139). Caffeine was the most commonly used (42/139, 30.2%), followed by alcohol (22/139, 15.8%), whereas 77 participants (55.4%) reported no substance use. No opioid use or performance‐enhancement drug use was reported among the footballers.

**Table 2:** Self‐reported substance use among study participants (*N* = 139).

Substance	Frequency (*n*)^a^	Prevalence (%)^b^
Alcohol	22	15.8
Caffeine	42	30.2
Cannabis	4	2.9
Tobacco	3	2.2
None	77	55.4

^a^ Some players used more than 1 substance; hence, the number of responses exceeds 139, i.e., 148.

^b^ Prevalence = number of participants choosing a substance/total number of participants.

### 3.2 Extent of Substance Use

Table 3 details the extent of substance use among participants using standard assessment tools, i.e., AUDIT, CUDIT‐R, and FTND tools. Low‐risk alcohol consumption as assessed by the AUDIT tool was reported by 18 study participants (18/139, 12.9%). This study found 95.5% of alcohol consumers had less than three drinks at a sitting. The commonest time for alcohol consumption was after matches (14/139, 10.1%), with off‐season use involving (7/139, 5.0%). Two participants (2/139, 1.4%) had their CUDIT‐R score in the low‐risk zone, and another two in the hazardous zone, while all tobacco users in this study also had an FTND score of 0, corresponding to low nicotine dependence.

**Table 3:** Pattern of substance use among participants as assessed by substance use assessment tools (*N* = 139).

Instrument	Frequency (*n*)	Percentage (%)
AUDIT		
No alcohol use	117	84.2
Low risk use	18	12.9
Hazardous use	4	2.9
Mean AUDIT score	3.6 ( ± 3.7)	
CUDIT‐R		
No cannabis use	135	97.1
Low risk use	2	1.4
Hazardous use	2	1.4
Mean CUDIT‐R score	6.3 ( ± 4.6)	
FTND		
No nicotine use	136	97.8
Low dependence	3	2.2
Mean FTND score	0	

Abbreviations: AUDIT, alcohol use disorders identification test, CUDIT‐R, cannabis use disorders identification test‐revised, FTND, Fagerström test for nicotine dependence, N, frequency.

Energy drinks were the most popular source of caffeine, accounting for 92.6% of caffeine intake (39/42 caffeine users), whereas 2 took coffee and 1 took tea. There was no energy binge drinking (taking more than 3 drinks per sitting) amongst the study participants. Only 2.2% (3/139) of participants were aware of performance‐enhancing drugs, although there was no reported use of such amongst the study participants.

### 3.3 Self‐Reported Motivators of Substance Use in Participants

Participants were asked to indicate as many reasons as applied, concerning reasons for substance use. The following reasons for use were given: alcohol for pleasure (68.1%), post‐match relaxation (18.2%), to improve low mood (9.0%), to cope with stress/pressure of football (4.5%); cannabis users indulged for pleasure (50.0%), stress coping (25.0%) and post‐match relaxation (25.0%); tobacco for pleasure (100.0%); and caffeine was mainly for energy (45.2%), pleasure (14.3%), post‐match relaxation (14.3%), and performance enhancement (11.9%).

### 3.4 Predictors of Substance Use

Table 4 illustrates associations between sociodemographic and footballing career characteristics and substance use in participants. Except for a significant negative association observed between the number of years spent at the current club and an increased odds of substance use (OR 0.81; 95% CI 0.65, 0.99; *p* = 0.042), there were no significant associations found with all other demographic and career characteristics.

**Table 4:** Predictors of substance use among study participants.

Characteristic	OR	95% CI	*p* value
Age	0.93	0.77, 1.13	0.500
Marital status			
Single	2.16	0.86, 5.58	0.100
In a relationship	—	—	—
Married	1.42	0.14, 13.1	0.800
Religion			
None	0.31	0.01, 8.77	0.400
Christianity	—	—	—
Islam	1.28	0.51, 3.29	0.600
Educational level			
No formal education	0.00	—	> 0.900
Junior high school	—	—	—
Senior high school	2.17	0.81, 5.95	0.120
Tertiary	2.31	0.53, 10.8	0.300
Source of income			
Football only	—	—	—
Football and other source	3.01	1.00, 9.71	0.055
Position played			
Goalkeeper	0.46	0.11, 1.78	0.300
Defender	—	—	—
Midfielder	0.73	0.27, 1.93	0.500
Forward	1.16	0.44, 3.05	0.800
Years spent playing professional football	0.90	0.68, 1.20	0.500
Years spent at current club	0.81	0.65, 0.99	0.042

Abbreviations: CI, confidence interval, OR, odds ratio.

## 4 Discussion

Prevalence of substance use was 44.6% with caffeine use being the highest (30.2%). Alcohol was the second most commonly used (15.8%), followed by cannabis and tobacco at 2.9% and 2.2% respectively. This is similar to a related study in amateur footballers, where substance use was at 49.7%, but most attributable to cannabis at 23%, followed by tobacco at 10% and opioids at 7.5% [24]. Another study in a sample comprised of both amateur and professional Cameroonian footballers found a 41% prevalence of alcohol. These results highlight the differences in the epidemiology of substance use, based on the profile of substances as well as the level of sports participation (professional *vs.* amateur) [23]. The disparity noted in these studies may stem from geographical and cultural differences, as well as variations in medical regulations across different levels of sports [4, 23].

Caffeine was the most commonly used substance, with a prevalence of 30.2%. Caffeine is not a controlled substance; it is socially accepted and widely marketed (as in coffee, energy drinks, caffeinated tea); hence, this finding may reflect ease of accessibility. Energy drinks were the preferred means of caffeine consumption (92.9% of all caffeine use and 28.1% of study participants). This rate (28.1%) is much lower than the reported 62.6% of energy drink use in Ghanaian university athletes [43]. This may be because participants in this study are professionals as compared to university athletes. This is corroborated by findings in a study in Lagos, Nigeria [25]. Excessive caffeine use is associated with severe dehydration when coupled with sweating, the development of acute and chronic headaches, and recognized mental disorders such as caffeine‐induced sleep disorder, anxiety, and intoxication [44, 45, 46]. In this study, however, no participant reported indulging in energy binge drinking. This low caffeine intake per sitting, therefore, decreases the likelihood that players will exceed the 400 mg/day caffeine intake threshold known to trigger adverse health effects [47].

Most of the 22 alcohol users in this study had an AUDIT score below 7, which indicates low‐risk alcohol consumption. No player reported alcohol dependence, a stark contrast to more than half of the participants meeting or exceeding the criteria for dependence on the AUDIT in a study in the general population in Northern Ghana [48]. The low‐risk alcohol dependence rate in this study (12.9%) seems consistent with the 6.8% estimate for problematic alcohol consumption amongst general Ghanaian youth [49], but differs from studies in Australia and Europe, where more than half of the athletes were classified as hazardous drinkers [50, 51, 52]. Extent of alcohol consumption may also be measured by binge drinking, i.e., consuming five or more standard drinks (for males) at a sitting [53]. This study found no binge drinkers, with the majority of alcohol consumers (95.5%) taking less than three drinks at a sitting. This differed from more than half of US college athletes found to be binge drinkers in a related study [54]. These Ghanaian players, therefore, appear to consume alcohol less commonly than other African players and at much lower levels than their Western counterparts. However, as regular but moderate alcohol consumption can still impair cardiovascular and liver health [55], and is associated with anxiety and stress in high‐pressure environments such as professional sports [56], athlete education and regular monitoring may be required to maintain these low levels of consumption.

There were four cannabis users in this study (2.9%), with half at hazardous use and the other half at low‐risk use. The prevalence is much lower than the 16.2% marijuana use estimate among Ghanaian youth [49]. According to the CUDIT‐R score, this may indicate an elevated risk of dependence on cannabis use. This finding may warrant, in addition to keen education and monitoring, the institution of early support for users to prevent progression from mild to severe.

Our study also found remarkably low use of nicotine (2.2%) with no nicotine dependence (FTND score of zero). The low tobacco use rate may reflect the picture in the general Ghanaian population, where estimates are also low, ranging from 3.8% to 9.1% [29, 57]. This low nicotine dependence is associated with a lower likelihood of suffering cardiorespiratory complications of smoking tobacco [58], a greater likelihood of quitting smoking [59], and a lower probability of having withdrawal symptoms [60].

Although sports participation may increase one's risk of use of certain substances [2, 3, 4], in other countries, these reported low rates of alcohol, tobacco, and cannabis use, coupled with the preference for caffeine (energy drinks), are not entirely surprising. Firstly, the players may be aware of the effects of these substances on their athletic performance, general health, and their ban status (cannabis). In addition, there may be societal influences that explain the low rates, as cannabis, tobacco, and excessive alcohol intake are typically frowned upon as deviant behavior in Ghanaian society; hence, participants may have carried such notions into professional sports. For this reason, energy drinks may be a safer and more acceptable alternative. In addition, authority figures such as coaches, captains, and team managers could have facilitated a non‐indulgence culture within the teams, which could have been taken up, leading to low use of alcohol, cannabis, and tobacco. In such a conservative environment, players may not engage in substance use for fear of punishment, stigma, or judgment. Qualitative studies to further investigate these possibilities may be needed to explain these findings. However, these low rates (of alcohol, cannabis, and tobacco, when compared to caffeine) may also be due to underreporting to appear socially acceptable. It may also be attributable to the interviewer‐administered format of the study, as participants may not have been comfortable with sharing personal information, although confidentiality and privacy had been assured.

There was no reported use of performance‐enhancing drugs such as anabolic androgens amongst participants in this study, lower than the 4.6% rate in high school athletes in Ghana [30], or the 3.9% rate in South Africa [61]. Although impressive, there was very low knowledge (2.2%) of these substances, similar to findings in Cameroon [20]. This is rather concerning, as a lack of knowledge may lead to inadvertent use and increased odds of high‐risk use. In addition, performance‐enhancing drugs are some of the most commonly used and tested substances in sports, and it would therefore be expected of professional athletes to be knowledgeable about them. These findings reflect a gap in substance use education and training in professional football and underscore the urgent need for anti‐doping education.

Different substances may be used for a variety of reasons, and the effects reflect those differences. Energy was the main reason for caffeine use (45.2% of users). Research into the effects on energy supply and athletic performance supports these motivating factors [19]. The major reason given by alcohol users for their consumption was recreational use and post‐match relaxation. This reason is connected to the most common time of alcohol intake being after matches, aligning with observations elsewhere [62]. Pleasure was also mentioned in tobacco and cannabis use. This pattern reflects broader societal trends of pleasure‐seeking behavior, highlighting that professional players are not exempt from society's tendency toward hedonism [63], as well as the popularity of these substances purely for recreational purposes rather than for performance enhancement [4].

No significant associations were observed between substance use and demographic or professional variables such as age, education, or playing position. However, longer tenure at the current club was inversely associated with substance use, suggesting that organizational stability and team cohesion may protect against risky behaviors. Players who remain longer with a club may benefit from stronger support systems, consistent health routines, and reduced stress, whereas frequent transfers could increase vulnerability to maladaptive coping strategies.

As a cross‐sectional study, the findings only reflect substance use at the time of data collection, which cannot accurately capture changing substance use behaviors. Self‐reporting of substance use introduces risks of underreporting and social desirability bias, as admitting to use may threaten players' professional and reputational standing. Therefore, employing objective measures of substance use, such as biological testing, could help generate more accurate data. Generalizability might also be improved by using a probability sampling method and including the full roster of teams and their registered players, which could reduce response bias. However, as the first study on substance use among professional footballers in Ghana and one of the few in Africa, this research highlights the burden and substances used, identifies priority areas for systemic change, and adds valuable information to the scarce scientific literature on African sports. In addition to exploring reasons behind substance use, assessing the extent of use through standardized tests like AUDIT provides a clearer picture of substance use among footballers compared to similar studies in other African countries. This is important because mere substance use does not necessarily indicate dependence, and different levels of use require distinct management strategies.

## 5 Conclusions

This study found low levels of substance use among professional footballers, except for high caffeine consumption, particularly from energy drinks. Reported use and dependence on alcohol, cannabis, and nicotine were minimal. These patterns may reflect athletes' commitment to healthy lifestyles, structured routines, and the professional and social support systems surrounding competitive sports. However, the use of non‐probability sampling and potential underreporting due to social desirability or fear of sanctions should be considered when interpreting the findings.

Future studies should incorporate biological testing to validate self‐reported data. Sustained athlete‐focused interventions, including health education and substance use counselling, highlighting their implications for their physical, mental, and athletic performance, are essential to maintain low prevalence and prevent escalation to harmful use. The National Anti‐Doping Committee under the Ministry of Youth and Sports in Ghana and the Medical Committee of the Ghana Football Association represent valuable resources for partnership and oversight of such strategies. These can be achieved by partnering with team medical professionals, psychologists, and club management to deliver structured education, screening, and rehabilitation for players. For instance, these may be organized before tournaments or in the pre‐season period. Periodic surveys may also be employed throughout teams and leagues to track and predict substance use behavior to facilitate planning of appropriate support services.

Clubs should foster stable and supportive environments by promoting longer player tenure, regular health and mental well‐being assessments, and structured substance‐use education. Orientation and psychological support for newly transferred players may further mitigate risk. Additionally, encouraging healthier energy alternatives, such as carbohydrate–electrolyte solutions, protein supplements, or low‐caffeine beverages, and teaching stress management techniques, including mindfulness and sleep optimization, could help reduce reliance on psychoactive substances.

Overall, maintaining a culture of health promotion, education, and mental health support within clubs is key to sustaining clean and safe sports in Ghana.

## Author Contributions

**Isaac Odei:** Conceptualization, methodology, software, data curation, investigation, validation, formal analysis, writing – original draft, writing – review and editing, project administration. **Yaw Akye Essuman:** Data curation, formal analysis, writing – review and editing, software, investigation, validation. **Angela Lamensdorf Ofori‐Atta:** conceptualization, methodology, validation, writing – review and editing.

## Funding

The authors received no specific funding for this work.

## Ethics Statement

The study was approved by the University of Ghana Community Health Dissertation Review Committee (UGMS‐CHDRC/152/2024) and adhered to the tenets of the Declaration of Helsinki.

## Conflicts of Interest

The authors declare no conflicts of interest.

## Transparency Statement

The lead author Isaac Odei affirms that this manuscript is an honest, accurate, and transparent account of the study being reported; that no important aspects of the study have been omitted; and that any discrepancies from the study as planned (and, if relevant, registered) have been explained.

## Data Availability

The datasets generated during and/or analysed during the current study are available from the corresponding author on reasonable request.
